# Effect of a soft exosuit on daily life gait performance in people with incomplete spinal cord injury: study protocol for a randomized controlled trial

**DOI:** 10.1186/s13063-024-08412-2

**Published:** 2024-09-06

**Authors:** L. Visch, B. E. Groen, A. C. H. Geurts, I. J. W. van Nes, N. L. W. Keijsers

**Affiliations:** 1 Department of Research, Sint Maartenskliniek, Nijmegen, The Netherlands; 2 Department of Sensorimotor Neuroscience, Donders Institute for Brain, Cognition and Behavior, Radboud University, Nijmegen, The Netherlands; 3 Department of Rehabilitation, Donders Institute for Brain, Cognition and Behavior, Radboud University Medical Center, Nijmegen, The Netherlands; 4 Department of Rehabilitation, Sint Maartenskliniek, Nijmegen, The Netherlands

**Keywords:** Incomplete spinal cord injury, Exosuit, Gait, Ambulation, Home and community setting

## Abstract

**Background:**

People with incomplete spinal cord injury (iSCI) often have gait impairments that negatively affect daily life gait performance (i.e., ambulation in the home and community setting) and quality of life. They may benefit from light-weight lower extremity exosuits that assist in walking, such as the Myosuit (MyoSwiss AG, Zurich, Switzerland). A previous pilot study showed that participants with various gait disorders increased their gait speed with the Myosuit in a standardized environment. However, the effect of a soft exosuit on daily life gait performance in people with iSCI has not yet been evaluated.

**Objective:**

The primary study objective is to test the effect of a soft exosuit (Myosuit) on daily life gait performance in people with iSCI. Second, the effect of Myosuit use on gait capacity and the usability of the Myosuit in the home and community setting will be investigated. Finally, short-term impact on both costs and effects will be evaluated.

**Methods:**

This is a two-armed, open label, randomized controlled trial (RCT). Participants will be randomized (1:1) to the intervention group (receiving the Myosuit program) or control group (initially receiving the conventional program). Thirty-four people with chronic iSCI will be included. The Myosuit program consists of five gait training sessions with the Myosuit at the Sint Maartenskliniek. Thereafter, participants will have access to the Myosuit for home use during 6 weeks. The conventional program consists of four gait training sessions, followed by a 6-week home period. After completing the conventional program, participants in the control group will subsequently receive the Myosuit program. The primary outcome is walking time per day as assessed with an activity monitor at baseline and during the first, third, and sixth week of the home periods. Secondary outcomes are gait capacity (10MWT, 6MWT, and SCI-FAP), usability (D-SUS and D-QUEST questionnaires), and costs and effects (EQ-5D-5L).

**Discussion:**

This is the first RCT to investigate the effect of the Myosuit on daily life gait performance in people with iSCI.

**Trial registration:**

Clinicaltrials.gov NCT05605912. Registered on November 2, 2022.

**Supplementary Information:**

The online version contains supplementary material available at 10.1186/s13063-024-08412-2.

## Introduction

### Background/rationale

An incomplete spinal cord injury (iSCI) is caused by partial damage to the spinal cord and can lead to loss of motor, sensory, and autonomic functions, depending on the spinal level and severity of the lesion [1]. An iSCI can be caused by trauma, inflammation, vascular damage, or a neoplastic or degenerative process [1]. The estimated prevalence is 223–755 per million people worldwide [2, 3] with approximately 8000 people in the Netherlands [4]. People with iSCI often have decreased gait capacity, which limits their ability to ambulate in the home and community setting [5–7]. According to the World Health Organization’s International Classification of Functioning [8], gait capacity refers to what people are able to do in a standardized environment and daily life gait performance refers to what people habitually do in daily life (i.e., in the home and community setting). As a consequence of decreased gait capacity, people with iSCI are at risk of developing a sedentary lifestyle resulting in a vicious circle of declining daily life gait performance and gait capacity. Furthermore, a sedentary lifestyle increases the risk of secondary complications such as cardiovascular disease [9], muscle contractures [10], pressure sores [10], depression [11], chronic pain [11], and bowel problems [12]. This, in turn, puts them at an even higher risk to become inactive [13]. Conversely, increased gait capacity and daily life gait performance are related to improved health, more independence, better participation to society, and increased quality of life [14, 15].

To enhance ambulation in the home and community setting for people with spinal cord injury (SCI), assistive technology presents a compelling solution. Among the available options, powered assistive devices such as wearable exoskeletons offer significant potential. These devices use rigid structures to compensate for the loss of leg muscle strength [16] and are specifically designed for people with complete spinal cord injury who are otherwise wheelchair bound [17]. However, exoskeletons have notable limitations, e.g., their heavy weight (ranging from 13 to 48 kg) and their limited walking speed (0.1 m/s to 0.4 m/s) [18, 19]. Moreover, most exoskeletons completely take over the control of the lower limbs, irrespective of the contribution from active leg muscles [18]. Consequently, rigid exoskeletons are not well suited for people with iSCI who possess residual leg muscle strength and gait capacity.

A new breed of devices, known as soft exosuits, has been developed to assist neurological patients with residual leg muscle strength and gait capacity during walking [18]. A soft exosuit transmits forces across the lower-limb joints and consists of soft textiles with a minimum of rigid structures, making it light-weighted, but it requires substantial voluntary contribution from active leg muscles [20]. Exosuit designs vary from unilateral to bilateral and deliver mechanical support in different ways, e.g., ankle plantarflexion, knee extension, and/or hip extension [18, 21, 22]. One type of exosuit is the Myosuit, a commercially available bilateral exosuit providing assistance to knee and hip extension [23]. A pilot study involving participants with various neurological disorders has shown an increased gait speed while using the Myosuit compared to baseline gait speed measured in a standardized environment [18]. This study also showed that five training sessions of 45 min each was safe and feasible. There was a relatively high adherence to the study protocol and no adverse events were reported [18]. In addition, a case study with a person with iSCI has shown an increase in walking speed and walking efficiency with the Myosuit [24].

Currently, exoskeletons and exosuits are mainly used as gait re-training devices. Only one study showed that the Myosuit is feasible to be used for various activities in the home and community setting by people with leg muscle weakness [25]. However, to improve ambulation in the home and community setting in people with iSCI, the assistive effect of a soft exosuit on daily life gait performance needs to be investigated.

### Objectives

The primary study objective is to examine the assistive effect of a soft exosuit (Myosuit) on daily life gait performance in people with iSCI. The second objective is to investigate the effect of Myosuit use on gait capacity and the usability of the Myosuit in the home and community setting. Finally, short-term impact on both costs and effects will be evaluated. It is hypothesized that the assistive effect of the Myosuit increases daily life gait performance and that Myosuit use increases gait capacity in people with iSCI. It is also expected that its usability is satisfactory.

## Methods

### Regulation statement and ethics approval

The study is approved by the internal review board of the Sint Maartenskliniek and the regional medical ethics committee Oost-Nederland (2022–13719, NL80641.091.22). Any modifications of the protocol will be notified via an amendment. The trial has been registered on Clinicaltrials.gov (NCT05605912). This study will be conducted in accordance with the Declaration of Helsinki (64th WMA General Assembly, Fortaleza, Brazil, October 2013) and the Medical Research Involving Human Subjects Act. This protocol is reported according to the SPIRIT guidelines rand checklist [26]. All items of the WHO Trial Registration Data Set can be found in the protocol [27].

### Study design and setting

**Fig. 1:**
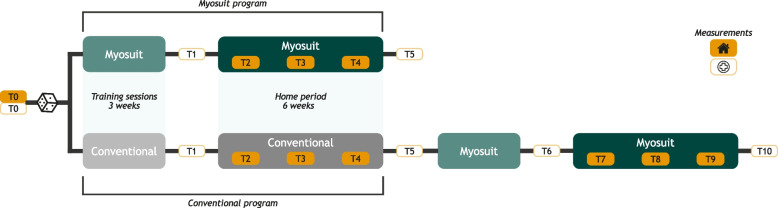
Schematic overview of the study design

This study is conducted at the Sint Maartenskliniek within the Gait Expertise Center, Nijmegen, The Netherlands. When the inclusion of participants is insufficient, the possibility of including another rehabilitation center will be considered. A patient representative was involved during the design phase of the study. It is a single-center, two-armed, open-label randomized controlled trial (RCT) which is visualized in Fig. 1. For this study, a superiority framework is used. To achieve a valid evaluation of the effect of the Myosuit on daily life gait performance, a control group is added to the study design to control for bias due to non-specific effects of attention and training in the intervention group.

### Recruitment

Potential participants who meet the eligibility criteria will be recruited by rehabilitation physicians working in the outpatient clinic at the Sint Maartenskliniek. Alternatively, interested patients may apply for the study themselves by contacting one of the rehabilitation physicians or the researchers. All individuals expressing interest in the study and granting permission to share their contact details will be contacted by the primary researcher (LV), who will send the information letter. After 1 week, the primary researcher will follow-up with the patients to address any questions or concerns they may have regarding the information provided. If patients indicate that they are willing to participate, the primary researcher will verify their eligibility. In the case a patient has applied him/herself, his/her eligibility will be verified by one of the rehabilitation physicians as well. The primary researcher (LV) will obtain informed consent at the baseline visit. Participants can leave the study at any time for any reason if they wish to do so without any consequences.

### Eligibility criteria

#### Inclusion criteria

Participants must meet all of the following inclusion criteria:

- Spinal cord injury grade C or D according to the American Spinal Injury Association (ASIA) impairment scale,
- At least 6 months after injury,
- Age ≥ 18 years,
- Sufficient hand function to don and doff the Myosuit or having a caregiver who is available to help donning and doffing the Myosuit at home,
- Reduced gait capacity due to reduced knee and/or hip strength (Medical Research Council (MRC) scale < 5),
- Able to stand up from a chair without deviating to the left or right side more than 45° during the rising movement,
- Able to independently ambulate for 10 m with or without assistive devices,
- Having a personal aim to improve walking distance, speed, or gait capacity otherwise.

#### Exclusion criteria

If participants meet any of the following criteria, they will not be eligible:

- Another (neurological) disease that may influence motor performance,
- Wounds that can be worsened by wearing the Myosuit,
- Body height < 150 cm or > 195 cm,
- Body weight < 45 kg or > 110 kg,
- Pregnancy,
- Flexion contracture at the knee or hip joint > 10°,
- Varus or valgus deformity at the knee > 10°.

### Allocation

Participants will be randomly allocated to either the intervention group (receiving the Myosuit program) or the control group (initially receiving the conventional program) using a block randomization method. After completion of the conventional program, the control group will as yet be enrolled in the Myosuit program (see Fig. 1). Randomization will be stratified based on preferred walking speed as assessed by the 10-Meter Walk Test (2 categories: < 0.6 m/s; ≥ 0.6 m/s). Group allocation will be done using a 1:1 ratio and in blocks with variable block sizes. Randomization will be performed by the primary researcher (LV), who is aware of the method of the group allocation. CastorEDC, a data management system for academic studies (www.castoredc.com), is used for the randomization.

### Blinding

Given the nature of the intervention, blinding of participants or treating physical therapists is not feasible. Because the primary researcher (LV) is responsible for the randomization process, the certification procedure of the Myosuit for home use, and for all measurements, outcome assessment cannot be blinded. The statistical analyses will not be blinded because they have been defined beforehand.

### Procedure

At T0 (see Fig. 1), baseline assessments of daily life gait performance, gait capacity, and quality of life will be conducted. Subsequently, participants will be randomized to the intervention group (receiving the Myosuit program) or control group (initially receiving the conventional program).

Participants in the intervention group receive the Myosuit program which consists of five gait training sessions (in 3 weeks) with the Myosuit at the Sint Maartenskliniek. Thereafter, questionnaires regarding the usability will be delivered (T1). In addition, participants will perform a test to receive a certificate for using the Myosuit themselves at home. A 6-week home period follows, during which participants have access to the Myosuit. Measurements of daily life gait performance will be conducted during the first, third, and sixth week of the home period (T2, T3, T4). After the 6-week home period, measurements of gait capacity, usability, and quality of life will be taken at the Sint Maartenskliniek (T5).

Participants in the control group will receive four gait training sessions (in 2 weeks) at the Sint Maartenskliniek, followed by a 6-week home. Measurements of daily life gait performance will be conducted during the first, third, and sixth week of the home period (T2, T3, T4). After the 6-week home period, measurements of gait capacity and quality of life will be taken at the Sint Maartenskliniek (T5). Thereafter, the control group will as yet be enrolled in the Myosuit program which will be identical to the Myosuit program of the intervention group. Usability questionnaires will be delivered at T6 and daily life gait performance will be assessed during the first, third, and sixth week of the home period (T7, T8, T9). Measurements of gait capacity, usability, and quality of life will be taken at T10.

### Interventions

#### Myosuit program

Participants in the intervention group receive the Myosuit (MyoSwiss AG, Zurich, Switzerland) for use as an assistive device at home and in the community for 6 weeks. The Myosuit is a soft exosuit that provides antigravity support to the user’s knee and hip joints while standing and walking [16]. This light-weight wearable device (5.5 kg) supports the self-initiated movements while walking without taking over the motor control of the lower limbs [18]. Before the home period, participants receive a flyer with general exercise recommendations tailored to people with SCI. Throughout the home period, participants fill out a logbook every day recording whether and for which activities they use the Myosuit.

Before using the Myosuit at home, participants receive five individual gait training sessions at the Sint Maartenskliniek. These sessions are led by physical therapists who are experienced in treating people with SCI and who have successfully completed a Myosuit certification course. Each training session consists of two parts. During the first part (30 min per session), participants receive instructions on how to use the Myosuit independently at home. During the second part (30 or 60 min per session), participants perform functional exercises with the Myosuit, based on the FIT-Stroke exercises [28], including sit-to-stand and stand-to-sit movements, sideways movements with and without transferring objects across a distance of 3 m, balance exercises including reaching movements, stepping up and down, walking at comfortable and maximum speed, ascending and descending stairs, completing an obstacle course, and walking outside. The total training time of the second training session parts is 4 h. As for the gait training sessions, physical therapists are instructed to relate the exercises to the daily activities that participants are able to perform with the Myosuit in their home and in the community.

#### Conventional program

Participants assigned to the control group will receive four individual gait training sessions at the Sint Maartenskliniek, in which they perform the same functional exercises as the intervention group (60 min per session). The total training time is 4 h. The physical therapists are instructed to relate the exercises performed during the training sessions to the daily activities of the participants. After the training sessions, a 6-week home period follows. Similar to the intervention group, participants receive a flyer with exercise recommendations tailored to people with SCI and they will keep a logbook recording their weekly physical activities.

### Assessments

All assessments will be performed following standard procedures and conducted by the primary researcher (LV) who has been trained to perform all assessments. During all assessments, participants are allowed to use assistive devices such as walking aids and/or orthoses. If participants use an assistive device during any test, this will be documented and kept constant throughout subsequent assessments. Table 1 shows the assessments at different timepoints.

**Table 1:** Standard protocol items: SPIRIT figure (summary of participant timeline)

	**Enrolment**	**Allocation**	**Post allocation**				**Close out RCT**	Post allocation*				Close out*
**Timepoint**	-T_1_	T0	T1**	T2	T3	T4	T5	T6*	T7*	T8*	T9*	T10*
**Enrolment**												
Eligibility screen	**x**											
Informed consent	**x**											
Allocation		**x**										
**Assessments**												
*Primary outcome measures*												
Walking time per day		**x**		**x**	**x**	**x**			**x**	**x**	**x**	
*Secondary outcome measures*												
Gait quality		**x**		**x**		**x**			**x**		**x**	
PWS		**x**					**x**					**x**
MWS		**x**					**x**					**x**
6MWT		**x**					**x**					**x**
SCI-FAP		**x**					**x**					**x**
SUS			**x**				x**	**x**				**x**
D-QUEST			**x**				x**	**x**				**x**
EQ-5D-5L		**x**					**x**					**x**
GDSSeS		**x**					**x**					**x**
*Other outcome measures*												
Demographics		**x**										

*Abbreviations*: *PWS *preferred walking speed, *MWS *maximum walking speed, *6MWT *6-Minute Walk Test, *SCI-FAP *Spinal Cord Injury Functional Ambulation Profile, *SUS *System Usability Scale, *D-QUEST *Dutch version of the Quebec User Evaluation of Satisfaction with assistive Technology, *EQ-5D-5L *European Quality of Life Five Dimension; *GDSSeS *General and Disease Specific Self-efficacy Scale, *-T*_*1 *_enrolment, *T0 *baseline, *T1 *questionnaires for the intervention group only, *T2 *first week of the home period, *T3 *third week of the home period, *T4 *sixth week of the home period, *T5 *after the intervention, *T6 *questionnaires for the control group only, *T7 *first week of the home period for the control group only, *T8 *third week of the home period for the control group only, *T9 *sixth week of the home period for the control group only, *T10 *after the intervention for the control group only

^*^Only applicable for the control group

^**^Only applicable for the intervention group

#### Primary outcome

The primary outcome measure for daily life gait performance is average walking time per day (min). It will be assessed with the Activ8 sensor (Activ8 Basic Activity Tracker, Activ8, Valkenswaard, The Netherlands), a single-unit activity monitor based on triaxial accelerometry [29]. The Activ8 will be placed on the participants’ right upper leg and measures lying, sitting, standing, walking, running, and cycling activities. Data sampling frequency is 50 Hz. The output provides information on the duration of specific activities within 1 min. The Activ8 has been validated in healthy individuals [29] and post-stroke patients [30] to assess the above-mentioned activities during daily life. Participants will wear the Activ8 continuously for seven consecutive days, 24 h a day. Baseline data (T0) will be averaged over a 7-day period to calculate the mean total walking time per day. During the 6-week home period, data from T2, T3, and T4 (the first, third, and sixth week of the home period) and data from T7, T8, and T9 (the first, third, and sixth week of the home period) will be averaged across all days (T2–T4; T7–T9) to calculate mean total walking time per day. For the primary analysis, the between-group difference in change of mean walking time per day at T2–T4 versus T0 will be of main interest.

#### Secondary outcomes

##### Spatiotemporal gait characteristics

Secondary outcome measures of daily life gait performance are walking speed (m/s), step length (m), maximum gait bout length (steps), and double support phase duration (s) assessed with inertial measurement units (IMUs). Two IMUs will be attached to both feet, and one will be positioned at the sacrum for 3 days. A self-developed algorithm comparable to previously published algorithms [31–33] will be used to calculate the various gait parameters. Spatiotemporal gait characteristics will be averaged over a 3-day period to calculate the mean at T0 (baseline). Data from the 6-week home period at T2 and T4 (first and sixth week of the home period) and at T7 and T9 (first and sixth week of the home period) will be averaged across all days (T2 + T4; T7 + T9). The between-group difference in change of spatiotemporal gait characteristics at T2 + T4 versus T0 will be analyzed.

##### Preferred walking speed (PWS)

PWS (m/s) will be assessed with the 10-Meter Walk Test (10MWT) using a static start [34]. Participants will be instructed to walk comfortably across the 10-m distance, and the time taken will be measured with a stopwatch. Participants will perform the test three times (if feasible based on their gait capacity), and the average score will be calculated. PWS will be measured at T0 (baseline), T5, and T10 (after the intervention). The between-group difference in change of mean PWS at T5 (after the intervention) versus T0 (baseline) will be analyzed. The 10MWT is a valid and reliable measure for assessing gait capacity in people with iSCI [35].

##### Maximum walking speed (MWS)

MWS (m/s) will be assessed using the 10MWT with a static start [34]. Participants will be instructed to walk across the 10-m distance as fast as possible while maintaining safety. Time required to walk 10 m will be measured with a stopwatch. Participants perform the test three times (if feasible based on their gait capacity), and the average score will be calculated. MWS will be measured at T0 (baseline), T5, and T10 (after the intervention). The between-group difference in change of mean MWS at T5 (after the intervention) versus T0 (baseline) will be analyzed.

##### Walking distance (6MWT)

Participants will be instructed to walk as far as possible during a 6-min Walk Test (6MWT) [36]. They are allowed to rest if needed, but encouraged to continue walking when possible. Participants walk in a rectangle of 4 by 18 m. Perceived exertion will be assessed before and after the 6MWT using the BORG scale (scores ranging from 6 to 20) [37]. The outcome measure of the 6MWT is walking distance (m). Walking distance will be measured at T0 (baseline), T5, and T10 (after the intervention). The between-group difference in change of walking distance at T5 (after the intervention) versus T0 (baseline) will be analyzed. The 6MWT is a valid and reliable measure to assess gait capacity in people with iSCI [35].

##### Spinal cord injury functional ambulation profile (SCI-FAP)

The SCI-FAP is a multidimensional validated measure of gait capacity in people with iSCI [38]. It consists of seven items that are characteristics of daily life walking: walking and carrying a bag, the timed Up & Go test, walking on a carpeted area, avoiding obstacles, walking stairs, walking over a step, and walking through a door. Each task will be timed (s) and performed at comfortable walking speed using an assistive device (if needed) according to the protocol. The level of assistance needed will be assessed on a scale from 1 to 6 (scores ranging from “independent”, “one cane/crutch/rail”, “two canes/crutches/rails”, “walker”, “assistance from one person”, to “unable to complete the task”). The score for each item will be calculated by multiplying the time taken with the assistance needed. Each item is then normalized to the mean time of healthy individuals. Normalized scores for each item are summed. A low total score reflects better gait capacity. The SCI-FAP will be performed at T0 (baseline), T5, and T10 (after the intervention). The between-group difference in change of SCI-FAP score at T5 (after the intervention) versus T0 (baseline) will be analyzed.

##### The Dutch version of the system usability scale (D-SUS)

The D-SUS is a questionnaire comprising 10 items designed to evaluate the usability of a system [39]. Each item is scored on a 5-point Likert scale from “strongly disagree” to “strongly agree.” Item scores are converted to a total score ranging from 0 to 100, with a higher score indicating greater usability. A total score from 0 to 50 indicates “not acceptable”, from 51 to 67 indicates “marginal usability”, and from 68 to 100 indicates “acceptable usability.” The D-SUS will be delivered at T1 (after the Myosuit training sessions) and T5 (after the intervention) for the intervention group and at T6 (after the Myosuit training sessions) and T10 (after the intervention) for the control group. The D-SUS has been tested for its validity and reliability [39].

##### The Dutch version of the quebec user evaluation of satisfaction with assistive technology (D-QUEST)

The D-QUEST is a 12-item questionnaire that assesses user satisfaction with an assistive device (8 items), service provided (4 items), and overall satisfaction (average of 12 items) [40]. Each item is scored from 1 (“totally dissatisfied”) to 5 (“very satisfied”). A higher score indicates greater satisfaction. An item score of 4 and 5 will be considered as “satisfactory” [41]. In addition, the importance of each item is indicated by the frequency with which it has been indicated as one of the three most important items [41]. The D-QUEST will be delivered at T1 (after the Myosuit training sessions) and T5 (after the intervention) for the intervention group and at T6 (after the Myosuit training sessions) and T10 (after the intervention) for the control group. The D-QUEST has been tested for its validity and reliability [40].

##### Quality-adjusted life years (QALY) gain and costs

The European Quality of Life Five Dimension (EQ-5D-5L) will be used to calculate gained QALYs [42, 43]. The EQ-5D-5L is a questionnaire that measures a patient’s health state across five dimensions: mobility, self-care, usual activities, pain/discomfort, and anxiety/depression. Scores on each dimension range from 1 to 5, with a higher score indicating a better health state. The EQ-5D-5L will be delivered at T0 (baseline), T5, and T10 (after the intervention). Measurements at T0 (baseline) and T5 (after the intervention) will be analyzed. In addition, actual costs associated with the intervention (Myosuit program) will be calculated, including costs related to providing physical therapy, the Myosuit purchase costs, and the Myosuit maintenance costs. Costs associated with the conventional program are costs related to providing physical therapy. The costs for physical therapy will be calculated based on the average wages of current rehabilitation physiotherapy professionals in The Netherlands indicated by the Dutch Health Care Institute.

##### General and disease specific self-efficacy scale (GDSSeS)

The GDSSeS is a 10-item questionnaire designed to assess general self-confidence and confidence in self-managing one’s physical status. For this study, six items are added to assess self-confidence in walking. Each item is scored from 0 to 10, with higher scores indicating greater confidence. A total score is calculated by summing up the scores for all items. The GDSSeS will be delivered at T0 (baseline), T5, and T10 (after the intervention). Measurements at T0 (baseline) and T5 (after the intervention) will be analyzed.

#### Demographics

The following demographic data will be collected: sex, age (years), time since spinal cord injury (months), body mass index (BMI), spinal injury level according to the ASIA impairment scale [44], level of spinal cord injury, leg muscle strength assessed with the MRC scale [45], leg muscle tone assessed with the Ashworth Scale [46], and somatosensation of the lower limbs assessed with the ASIA impairment scale [44].

### Data management

The collected data will be entered in CastorEDC (www.castoredc.com), where each participant will receive a unique anonymous code. A number is assigned to the order of inclusion. The list with unique codes will be stored separately from the collected data. All authors will have access to the final dataset in CastorEDC until they are no longer involved in the study. All data will be stored for 15 years after the study has ended. An independent monitor is designated to the study and will conduct monitoring according to the Netherlands Federation of University Medical Centers (NFU) regulations [47] for “negligible risk intensity”, independent of investigators and sponsors. No other groups are involved in the trial oversight.

### Assessment of adherence and co-interventions

To enhance adherence to the study protocol, all training sessions and assessments are planned at baseline and participants have direct contact with the primary investigator (LV) about these dates during the study period. Adherence to the training protocol will be logged by the physical therapists. If participants will cancel a training session, an additional session will be scheduled. As for the 6-week home period, home use instructions will be given after the last training session, but participants will only have contact with the primary researcher regarding the assessments or in the case there is a technical problem with the Myosuit. Participants are asked to complete all assessments independent of how often the Myosuit is used at home. There are no restrictions with respect to usual care during the study period.

### Safety and adverse event reporting

The classification risk is estimated as “negligible risk” according to the NFU regulations [47]. According to protocol, adverse events will be logged and serious adverse events will be reported through the web portal ToetsingOnline to the accredited medical ethics committee.

### Sample size

An improvement of 500 steps per day is considered clinically relevant, corresponding to a 33% improvement of a baseline with 1500 steps per day [48]. Based on a stride time of 1.07 s, 500 steps per day corresponds to an increase of 4.5 min walking time per day [49]. For each group, a sample size of *N* = 14 is needed to demonstrate a difference in improvement of 500 steps per day between groups (SD = 400, *α* = 0.05, *β* = 0.10) [48]. Three patients will be added to each group (*N* = 17) allowing an 18% attrition rate; therefore, we aim to include a total of 34 participants. Sample size calculation is based on conducting analysis of variance (ANOVA) due to the absence of data for calculating a correlation that would be required for sample size calculation based on analysis of covariance (ANCOVA). Generally, statistical power of ANCOVA is higher than that of ANOVA.

### Statistical methods

For both the primary and secondary outcomes, an intention-to-treat analysis will be performed. For missing data, listwise deletion will be used. Additionally, the last observation carried forward method will be used in the sensitivity analysis.

#### Primary outcome

To indicate group differences in walking time per day (average values of the first, third, and sixth week of the home period, e.g., T2–T4), an ANCOVA will be performed using the pre-intervention values (T0) as covariates.

A secondary analysis will be performed on walking time per day for the combined Myosuit home use periods (average values of the first, third, and sixth week of the home period (T2-T4) versus pre-intervention values (T0) for the intervention group and average values of the first, third, and sixth week of the home period (T7-T9) versus pre-intervention values (T4) for the control group) to investigate whether specific personal characteristics are related to improvement resulting from Myosuit use. This analysis involves a regression model with the difference in minutes walking time per day as dependent variable and personal characteristics (such as lesion level, ASIA score, and age) as independent variables. No other analyses are preplanned.

#### Secondary outcomes

Group differences in gait capacity (PWS, MWS, 6MWT, SCI-FAP) after the intervention (T5) and spatiotemporal gait characteristics of daily life gait performance (walking speed, step length, maximum gait bout length, and double support phase duration) averaged over the first and sixth week of the home period (T2 and T4) will be tested with an ANCOVA using the pre-intervention values (T0) as covariates. Costs collected during the study will be specific Myosuit costs and training costs per patient for the intervention group and trainings costs per patient for the control group. Total QALYs will be assessed by the area under the curve. Short-term impact on both costs and QALYs will be analyzed by calculating the between-group differences in average costs per patient and average QALYs between T5 (after the intervention) and T0 (pre-intervention values). Cost differences will be expressed per costing category (average Myosuit costs and training costs per patient) to ensure insight is provided with regard to the impact on individual costing parameters. The results of the D-QUEST, SUS, and GDSSeS will be evaluated by descriptive analyses (mean and standard deviation).

## Discussion

Ambulation in the home and community setting is limited in many people with iSCI [5]. It has been shown that the Myosuit is feasible to be used for various activities in the home and community setting by people with leg muscle weakness [25], but the effect of the Myosuit to increase ambulation in people with iSCI remains unknown. This RCT investigates the assistive effect of the Myosuit on daily life gait performance in people with iSCI. If the Myosuit proves to be effective in increasing physical activity, it may lead to a less sedentary lifestyle and a reduction of secondary complications after iSCI.

We deliberately chose to use a cross-over design for the control group, as we expect this will improve patient inclusion. It also provides a better opportunity to investigate whether personal characteristics are related to improved daily life gait performance with the Myosuit by combining data from both groups. In addition, during the 6-week home periods, participants purposely receive only general exercise recommendations. Although specific home training recommendations may be more effective to increase physical activity, we are primarily interested in the natural improvement of walking time per day as a result of Myosuit availability. Due to the nature of the intervention and as a consequence of study constraints, it is not feasible to blind participants, physical therapists, or the assessor, which may introduce assessment bias, but the primary outcome measure is based on objective recording of daily life gait performance, a measure insensitive to influence from the assessor. Moreover, all measurement procedures are standardized as much as possible to minimize assessor bias.

### Dissemination

The results of this study will be published in (inter)national scientific journals, presented on (inter)national conferences, and communicated to people with iSCI who participated in this study. Authorship will be determined according to NFU regulations [47].

## Availability of data and the full protocol

Metadata and analysis code will be made accessible through an online portal. The protocol is reported at ClinicalTrials.gov (NCT05605912).

### Trial status

Participant recruitment has started in October 2022. The protocol is version 2.0, dated June 16, 2022. At the moment, 23 participants are included. It is expected that the recruitment is finished in December 2024.

## Supplementary Information

Supplementary Material 1.

Supplementary Material 2.

Supplementary Material 3.

Supplementary Material 4.

## Abbreviations

10MWT: 10-Meter Walk Test
6MWT: 6 Minutes’ walk test
ANCOVA: Analysis of co-variance
ANOVA: Analysis of variance
ASIA: American Spinal Injury Association
BMI: Body mass index
D-SUS: Dutch version of the System Usability Scale
D-QUEST: Dutch version of the Quebec User Evaluation of Satisfaction with assistive Technology
EQ-5D-5L: European Quality of Life Five Dimension
GDSSeS: General and Disease Specific Self-efficacy Scale
IMU: Inertial measurement units
iSCI: Incomplete spinal cord injury
MRC: Medical Research Council
MWS: Maximum walking speed
NFU: Netherlands Federation of University Medical Centers
PWS: Preferred walking speed
RCT: Randomized controlled trial
SCI: Spinal cord injury
SCI-FAP: Spinal Cord Injury Functional Ambulation Profile
QALY: Quality-adjusted life years
