# Supplementary material for: Effect of a soft exosuit on daily life gait performance in people with incomplete spinal cord injury: study protocol for a randomized controlled trial
Source: Trials. 2024 Sep 6;25:592. doi: 10.1186/s13063-024-08412-2 (PMC11378477; doi:10.1186/s13063-024-08412-2)
Supplement: Supplementary file 3 — Supplementary Material 3. [file 13063_2024_8412_MOESM3_ESM.pdf]

Bijlage D: toestemmingsformulier proefpersoon

Behorende bij

Effectiviteit van de Myosuit voor het verbeteren van het lopen bij mensen met een incomplete dwarsleasie

- Ik heb de informatiebrief gelezen. Ook kon ik vragen stellen. Mijn vragen zijn goed genoeg beantwoord. Ik had genoeg tijd om te beslissen of ik meedoe.
- Ik weet dat meedoen vrijwillig is. Ook weet ik dat ik op ieder moment kan beslissen om toch niet mee te doen met het onderzoek. Of om ermee te stoppen. Ik hoef dan niet te zeggen waarom ik wil stoppen.
- Ik geef de onderzoeker toestemming om mijn specialist die mij behandelt te laten weten dat ik meedoe aan dit onderzoek.
- Ik geef de onderzoekers toestemming om mijn gegevens te verzamelen en gebruiken. De onderzoekers doen dit alleen om de onderzoeksvraag van dit onderzoek te beantwoorden.
- Ik weet dat voor de controle van het onderzoek sommige mensen al mijn gegevens kunnen inzien. Die mensen staan in deze informatiebrief. Ik geef deze mensen toestemming om mijn gegevens in te zien voor deze controle.
- Wilt u in de tabel hieronder ja of nee aankruisen?

Ik geef toestemming om mijn gegevens te bewaren om dit te gebruiken voor ander onderzoek, zoals in de informatiebrief staat.	Ja ☐	Nee☐
Ik geef toestemming om mij eventueel na dit onderzoek te vragen of ik wil meedoen met een vervolgonderzoek.	Ja ☐	Nee☐

- Ik wil meedoen aan dit onderzoek.

Mijn naam is (proefpersoon): ………………………………..

Handtekening: ……………………… Datum : __ / __ / __

Ik verklaar dat ik deze proefpersoon volledig heb geïnformeerd over het genoemde onderzoek.

Wordt er tijdens het onderzoek informatie bekend die die de toestemming van de proefpersoon kan beïnvloeden? Dan laat ik dit op tijd weten aan deze proefpersoon.

Naam onderzoeker (of diens vertegenwoordiger):………………………………

Handtekening:……………………… Datum: __ / __ / __

De proefpersoon krijgt een volledige informatiebrief mee, samen met een getekende versie van het toestemmingsformulier.
