# Supplementary material for: Effect of a soft exosuit on daily life gait performance in people with incomplete spinal cord injury: study protocol for a randomized controlled trial
Source: Trials. 2024 Sep 6;25:592. doi: 10.1186/s13063-024-08412-2 (PMC11378477; doi:10.1186/s13063-024-08412-2)
Supplement: Supplementary file 4 — Supplementary Material 4. [file 13063_2024_8412_MOESM4_ESM.pdf]

Attachment D: informed consent participant

Belonging to

Effectiveness of the Myosuit to improve gait in people with incomplete spinal cord injury

- I have read the information letter. I was also able to ask questions. My questions were answered adequately. I had enough time to decide if I wanted to participate.
- I understand that participating is voluntary. I also understand that I can decide not to participate in the study at any time. Or to leave the study. I do not have to explain why I want to leave the study.
- I give the researcher permission to inform my treating specialist that I am participating in this study.
- I give the researchers permission to collect and use my data. The researchers will only use my data to answer the research question of this study.
- I understand some people may have access to all my data to monitor the study. These people are mentioned in the information letter. I give these people permission to access my data to monitor the study.
- Would you please check 'yes' or 'no' in the table below?

I give consent to store my data for use in other research, as stated in the information letter.	Yes ☐	No ☐
I give consent that I may be contacted to ask if I would like to participate in a follow-up study.	Yes ☐	No ☐

- I want to participate in this study.

My name is (participant): ………………………………..
Signature: ……………………… Date : __ / __ / __

I declare that I have fully informed this participant about the study.

If any information arises during the study that may affect the participant's consent, I will inform the participant in time.

Name researcher (or its representative):……………………………….
Signature:……………………… Date: __ / __ / __

The participant will receive a complete information letter, along with a signed version of the informed consent
