# Supplementary material for: Effect of a soft exosuit on daily life gait performance in people with incomplete spinal cord injury: study protocol for a randomized controlled trial
Source: Trials. 2024 Sep 6;25:592. doi: 10.1186/s13063-024-08412-2 (PMC11378477; doi:10.1186/s13063-024-08412-2)
Supplement: Supplementary file 1 — Supplementary Material 1. [file 13063_2024_8412_MOESM1_ESM.pdf]

Ubbergen, 16th of August, 2024

Dear Editor,

We herewith submit our revised manuscript entitled: *'Effect of a soft exosuit on daily life gait performance in people with incomplete spinal cord injury: study protocol for a randomized controlled trial'* (TRLS-D-24-00436). We would like to thank the reviewer and editor for the time they spent in evaluating our paper and for their valuable comments and suggestions. We addressed all issues raised by the reviewer and the editor, as described below on a point by point basis.

- Item 12: Page 10 lines 429-449 - please clearly indicate time points for primary and secondary outcome measurements, as per SPIRIT guidance. T2-T4 and T7-T9 do not mean anything . I went to Table 1 for clarification but there was not any. Lines 280-282 are the same. There may have been some explanation on lines 216 but it is important that this is repeated as it is not intuitive.

Detailed explanations can be found in lines 209-228. In lines 280-282, we made adjustments to the text and added information about the time points:
"During the six-week home period data from T2, T3, and T4 **(the first, third, and sixth week of the home period)** and data from T7, T8, and T9 **(the first, third, and sixth week of the home period)** will be averaged across all days (T2-T4; T7-T9) to calculate mean total walking time per day."
In line with the primary outcome, we made adjustments to the text and added information about the time points for the secondary outcomes (lines 286-386).

Lines 431-456 have been adjusted for clarification:
"Primary outcome

To indicate group differences in walking time per day (average values of **the first, third, and sixth week of the home period, e.g.** T2-T4) an ANCOVA will be performed using the a pre-intervention values (T0) as covariates.

A secondary analysis will be performed on walking time per day for the combined Myosuit home use periods (**average values of the first, third, and sixth week of the home period (**T2-T4**)** versus **pre-intervention values (**T0**)** for the intervention group and **average values of the first, third, and sixth week of the home period (**T7-T9**)** versus **pre-intervention values (**T4**)** for the control group) to investigate whether specific personal characteristics are related to improvement resulting from Myosuit use. This analysis involves a regression model with the difference in minutes walking time per day as dependent variable and personal characteristics (such as lesion level, ASIA score and, age) as independent variables. No other analyses are preplanned.

Secondary outcomes
Group differences in gait capacity (PWS, MWS, 6MWT, SCI-FAP) **after the intervention (**T5**)** and spatiotemporal gait characteristics of daily life gait performance (walking speed, step length, maximum gait bout length, and double support phase duration) averaged over **the first and sixth week of the home period (**T2 and T4**)** will be tested with an ANCOVA using the pre-intervention values (T0) as covariates. Costs collected during the study will be specific Myosuit costs and training costs per patient for the intervention group and trainings costs per patient for the control group. Total QALYs will be assessed by the area under the curve. Short term impact on both costs and QALYs will be analyzed by calculating the between-group differences in average costs per patient and average QALYs between T5 **(after the intervention)** and T0 **(pre-intervention values)**. Cost differences will be

expressed per costing category (average Myosuit costs and training costs per patient) to ensure insight is provided with regard to the impact on individual costing parameters. The results of the D-QUEST, SUS, and GDSSeS will be evaluated by descriptive analyses (mean and standard deviation).''

- Item 16a: Line 196. Please remove "(N=2 or N=4)". This information should be on a need-to-know basis. To reduce predictability of a random sequence, details of any planned restriction (e.g. blocking) should be provided in a separate document that is unavailable to those who enrol participants or assign interventions. If recruitment has started and authors are aware that blocking information may have been known to those recruiting and assigning interventions then this should be stated.

We have removed "(N=2 or N=4)".
Recruitment has started and blocking information is known to those recruiting and assigning interventions, this information is added to the manuscript in the following sentence: "Randomization will be performed by the primary researcher (LV)**, who is aware of the method of the group allocation.**"

- Table 1 SPIRIT figure: The Timepoint -T1 to T10 is not clear -Please clarify. This was not included in the legend.

*Clarification of the time points are added to the abbreviations in the legend of Table 1: "***-T_1: enrolment; T0: baseline; T1: questionnaires for the intervention group only; T2: first week of the home period; T3: third week of the home period; T4: sixth week of the home period; T5: after the intervention; T6: questionnaires for the control group only; T7: first week of the home period for the control group only; T8: third week of the home period for the control group only; T9: sixth week of the home period for the control group only; T10: after the intervention for the control group only.**"

Kind regards, on behalf of all authors,

Lara Visch

Address for correspondence:

Sint Maartenskliniek

Department Research & Innovation

Hengstdal 3, 6574 NA Ubbergen, The Netherlands

P: 024-3272701 / E: la.visch@maartenskliniek.nl
